# Pharmacologically targeted NMDA receptor antagonism by NitroMemantine for cerebrovascular disease

**DOI:** 10.1038/srep14781

**Published:** 2015-10-19

**Authors:** Hiroto Takahashi, Peng Xia, Jiankun Cui, Maria Talantova, Karthik Bodhinathan, Wenjun Li, Emily A. Holland, Gary Tong, Juan Piña-Crespo, Dongxian Zhang, Nobuki Nakanishi, James W. Larrick, Scott R. McKercher, Tomohiro Nakamura, Yuqiang Wang, Stuart A. Lipton

**Affiliations:** 1 Neuroscience and Aging Research Center, Sanford-Burnham-Prebys Medical Discovery Institute, La Jolla, California 92307, USA; 2 Panorama Research, Inc., Sunnyvale, California 94089, USA; 3 Institute of New Drug Research, Jinan University College of Pharmacy, Guangzhou 510632, China; 4 Department of Neurosciences, University of California San Diego School of Medicine, La Jolla, California 92039, USA

## Abstract

Stroke and vascular dementia are leading causes of morbidity and mortality. Neuroprotective therapies have been proposed but none have proven clinically tolerated and effective. While overstimulation of *N*-methyl-d-aspartate-type glutamate receptors (NMDARs) is thought to contribute to cerebrovascular insults, the importance of NMDARs in physiological function has made this target, at least in the view of many in ‘Big Pharma,’ ‘undruggable’ for this indication. Here, we describe novel NitroMemantine drugs, comprising an adamantane moiety that binds in the NMDAR-associated ion channel that is used to target a nitro group to redox-mediated regulatory sites on the receptor. The NitroMemantines are both well tolerated and effective against cerebral infarction in rodent models via a dual allosteric mechanism of open-channel block and NO/redox modulation of the receptor. Targeted S-nitrosylation of NMDARs by NitroMemantine is potentiated by hypoxia and thereby directed at ischemic neurons. Allosteric approaches to tune NMDAR activity may hold therapeutic potential for cerebrovascular disorders.

Focal cerebral ischemia (stroke) and vascular dementia (due to multiple strokes in the microvasculature) represent leading causes of severe cognitive dysfunction and death worldwide^1^. While excessive stimulation of *N*-methyl-d-aspartate-type glutamate receptors (NMDARs) is acknowledged to be important in the etiology of cerebrovascular damage^2^,^3^, NMDAR antagonists were previously found to be either ineffective or clinically intolerable for cerebral ischemic insults in humans despite promising early results in animal models^4^,^5^,^6^. Many in ‘Big Pharma’ have thus made the tacit assumption that NMDARs are not ‘druggable’ for cerebrovascular insults. However, the possibility exists that rather than the target being intractable, the specific antagonists used were not adequately designed. Data presented here support the premise that the case for NMDAR antagonists should be revisited.

One important relatively new concept concerns the population of NMDARs that are excessively activated during cerebral ischemia and vascular dementia. Recent evidence supports the notion that under most conditions pathological activity is predominantly mediated by extrasynaptic NMDARs, whereas physiological synaptic NMDAR activity triggers neuroprotective molecular pathways in neurons^7^,^8^. One well-studied NMDAR antagonist in particular, memantine^9^, represents an uncompetitive/fast off-rate (‘UFO’) drug, which primarily affects extrasynaptic/tonically-activated NMDARs over synaptic/phasically-activated NMDARs^10^,^11^,^12^,^13^. In fact, memantine has shown promise not only in animal studies of stroke but also in a phase 2 human clinical trial for efficacy in vascular dementia^14^,^15^,^16^,^17^. Nonetheless, despite its approval by the FDA and EMA for moderate-to-severe Alzheimer’s disease, the drug has not been tested in additional advanced trials for ischemic disease because of its fairly modest effects. Here, we develop and characterize improved analogs of memantine, representing aminoadamantane nitrates that demonstrate both increased efficacy and safety in animal models of cerebral ischemia. Importantly, detailed demonstration of target selectivity, electrophysiological properties, and neurobehavioral effects of the lead candidate, designated YQW-036/NMI-6979 NitroMemantine^18^,^19^, provide a promising therapeutic profile. Early pharmacokinetic studies also indicate that this drug may represent a viable candidate for diseases related to cerebral ischemia.

We show here that NitroMemantines offer an advantage over memantine, at least in part, because the new drugs manifest a dual site of action at the NMDAR; accordingly, NitroMemantine performs in a superior fashion to memantine in treatment of a rat model of focal cerebrovascular disease. The memantine moiety, which preferentially blocks excessively open NMDAR-operated channels in neurons undergoing ischemic insult, is used to target an NO-generating group to the redox modulatory sites of those NMDARs; these redox sites then undergo S-nitrosylation to cause channel desensitization, resulting in a dual action of the NitroMemantine drugs via channel blockade coupled to allosteric redox modulation^11^,^18^,^20^. The results show that NitroMemantine offers significantly more protection than memantine against cerebral ischemic insults, while sparing a greater proportion of synaptic transmission. Perhaps most importantly, NitroMemantine is an example of an FDA-approved drug (memantine) that is used to target a second moiety to the same receptor in order to enhance efficacy of action. This principal may serve as a new platform for clinically-tolerated drug discovery in the CNS.

## Results

### Open-channel block and redox effects of NitroMemantines

**Figure 1:**
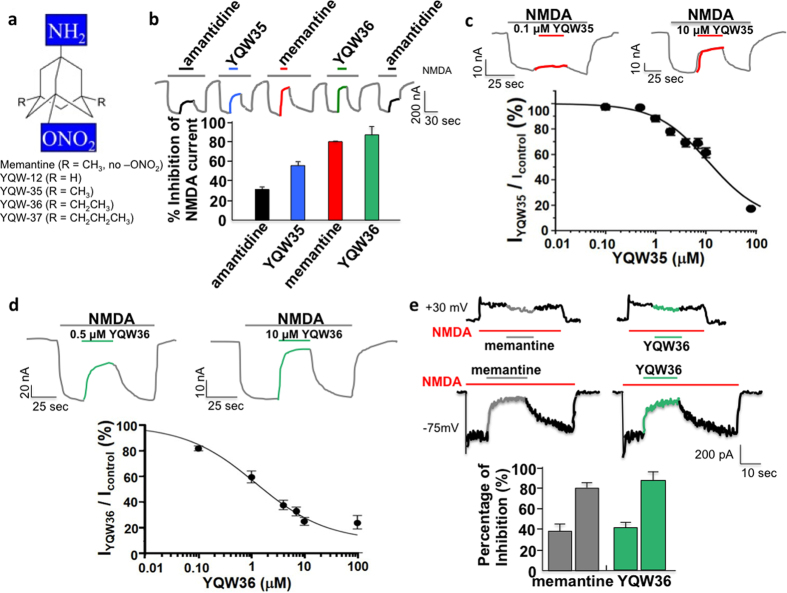
Channel block of NMDAR-mediated currents by various aminoadamantane drugs. (**a**) Structure of aminoadamantane and aminoadamantane nitrates. 1-Aminoadamantane hydrochloride (memantine) has methyl side chains (‘R’ groups) and does not contain a nitro group off the back end of the molecule. With addition of the nitro group for redox functionality, the affinity of the aminoadamantane moiety for the NMDAR-associated ion channel is diminished. Lengthening the ‘R’ side chains, however, compensates for this. While memantine and YQW-035 have methyl side chains, YQW-012 has only protons, YQW-036 has ethyl groups, and YQW-037, propyl groups. Not only does lengthening the side chains increase binding affinity in the channel, but also increases the lipophilicity and thus penetrance of the blood-brain-barrier, while decreasing aqueous solubility. (**b**) Amantadine (10 μM) inhibited less NMDA current at steady state than equimolar memantine. Equimolar NitroMemantine YQW-035 manifests less channel block than memantine but more than amantadine. In contrast, NitroMemantine YQW-036 steady-state channel block of approximately the same degree as memantine. Two-electrode voltage clamp of oocytes expressing recombinant GluN1/GluN2A receptors at a holding potential (Vh) −70 mV. Values are mean + s.e.m. (**c**) Dose-response of NitroMemantine YQW-035 at Vh = −70 mV. Values are mean ± s.e.m. (**d**) Dose-response of NitroMemantine YQW-036 at Vh = −70 mV. Values are mean ± s.e.m. (**e**) In primary rat cortical neurons, whole-cell patch clamp recordings revealed that 5 μM memantine or NitroMemantine YQW-036 produced an approximately equivalent degree of blockade at both –75 mV and less blockade at +30 mV, indicating the voltage-dependence of the block by the memantine moiety. In the histogram, for each data point, *n* ≥ 5 recordings per drug tested; left bar in each pair for responses at −75 mV and right bar for +30 mV responses. Values are mean + s.e.m.

We have previously shown that the NMDAR can be antagonized by memantine via open-channel block^10^,^14^ as well as by nitric oxide (NO)-based compounds via an allosteric redox-modulatory site(s) that is subject to S-nitrosylation^20^,^21^,^22^. Moreover, we demonstrated that memantine preferentially interacts with excessively (pathologically) open NMDAR-associated channels^11^,^13^. Additionally, during hypoxia, NO-based compounds preferentially react with allosteric cysteine residues in the NMDAR to limit excessive activity^22^. In an effort to combine these attributes and produce a dual functional drug that targets the NO group to pathologically-open NMDAR-coupled channels, we ‘piggybacked’ onto memantine an NO_x_ group (where x = 1 or 2). For this purpose, we initially synthesized several hundred NitroMemantine derivatives (and control scaffolds) in order to perform a detailed structure-activity relationship (SAR) of these novel drugs^19^. Of these, one series of drugs showed particular promise and possessed a nitro (−NO_2_) group opposite the bridgehead amine (−NH_2_), which is protonated (−NH_3_^+^) at physiological pH (Fig. 1a; see Supplementary Methods for details of chemical synthesis)^18^. We have previously shown that, via this bridgehead amine, memantine binds at or near the Mg^2+^ site of the NMDAR, particularly on the GluN1 (previously denoted as NR1) subunit^23^.

We have studied in detail a number of these NitroMemantine compounds, which utilize the high-affinity memantine binding site on NMDARs to target the NO_x_ group for interaction with the S-nitrosylation/redox site external to the memantine-binding site. In particular, we found that by adding the –ONO_2_ functional group of nitroglycerin onto memantine (represented by compound YQW-035), we decreased the potency of steady-state channel block during electrophysiological recordings from frog oocytes expressing recombinant NMDARs (Fig. 1b). We found that by lengthening the side chains of memantine we could compensate for lost drug affinity in the channel associated with the addition of the –ONO_2_ group. YQW-036 (with ethyl groups) represents the preferred-length of side chain, as YQW-037 (with propyl groups) cannot be dissolved in aqueous solutions. The IC_50_ of inhibiting NMDA-induced current for short-term additions of YQW-35 (which has methyl side groups similar to memantine) and YQW-36 (in which the side groups have been lengthened to ethyl) are 6.3 and 2.4 μM, respectively, when measured under steady-state conditions after short-term drug addition during voltage clamp recordings at a holding potential of −70 mV (Fig. 1c,d). These values can be compared to the IC_50_ of memantine (0.5–1 μM) and amantadine (~35 μM)^10^,^24^. Thus, the NitroMemantine drug designated YQW-036 manifests an affinity for the NMDAR-operated ion channel approaching that of memantine. While these values were obtained in the nominal absence of extracellular Mg^2+^, we have shown that YQW-036 also blocks NMDA-evoked currents in the presence of normal concentrations of Mg^2+^ (Supplementary Fig. 1).

Additionally, these new compounds, like memantine, display voltage-dependent inhibition of NMDA-evoked currents in recordings from rat cerebrocortical neurons, as expected for a positively-charged open-channel blocker (Fig. 1e). However, this property may partly account for the relatively weak clinical efficacy of memantine. When neurons become energetically compromised, and consequently depolarize because of the inrush of positively charged Na^+^ and Ca^2+^ ions^25^,^26^, memantine is repelled from the ion channel. Thus, the ‘sickest’ neurons, which need neuroprotection the most, become vulnerable.

**Figure 2:**
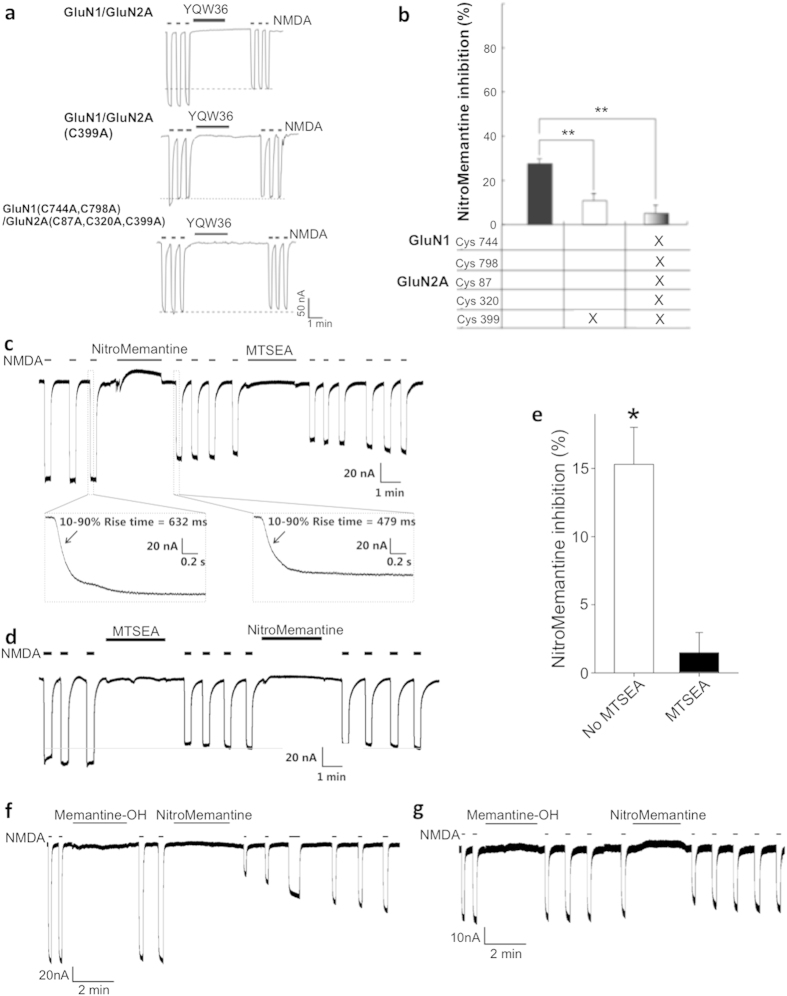
Redox effects of NitroMemantine YQW-036 mediated by S-nitrosylation. (**a**) Representative recordings under two-electrode voltage clamp in oocytes expressing wild-type (WT) or non-nitrosylatable cysteine mutant NMDAR subunits. (**b**) Quantification of contribution of cysteine residues in various NMDAR subunits to the inhibitory effect of S-nitrosylation (data are mean + s.e.m. for *n* ≥ 5 recordings per data point, **P < 0.01). (**c**) In oocyte recordings of NMDA-evoked currents under voltage clamp, inhibition by NitroMemantine (10 μM) largely occluded any additional effect of subsequent application of the sulfhydryl-reactive reagent MTSEA (0.5 mM), as expected if the same cysteine residues were involved. Inset: expanded time scale to show rise time of NMDA-evoked current before and after NitroMemantine addition. (**d**) Inhibition of NMDA-evoked currents by MTSEA occludes the redox-mediated inhibitory effect of subsequent addition of NitroMemantine. (**e**) Quantification of effects shown in c and d. Data are mean + s.e.m. for *n* ≥ 6 oocytes recordings per data point (*P < 0.005). (**f**) NitroMemantine inhibits NMDAR activity by S-nitrosylation. Addition of 100 μM NitroMemantine YQW-036, but not its metabolite memantine-OH, inhibited NMDA-evoked current in oocytes expressing GluN1/GluN2A NMDARs recorded under voltage clamp conditions used to observe S-nitrosylation (Vh = −60 mV; data representative of *n* ≥ 4 oocytes recordings). (**g**) Mutation of the memantine binding site in the GluN1 subunit of the NMDAR prevents the redox effect of NitroMemantine. Disruption of the memantine binding site by mutation GluN1(N616R) abrogated the redox-mediated activity of NitroMemantine (100 μM). Data representative of *n* ≥ 3 oocytes recordings.

In an attempt to remedy this shortcoming, the NitroMemantine adducts manifest a second, redox-based inhibition of NMDA-induced current. This additional allosteric site of action produces a relatively long-lasting inhibition of NMDARs resulting from S-nitrosylation that is mediated by the nitro group, as demonstrated in voltage-clamped oocyte recordings (Fig. 2a)^20^,^27^,^28^. Unlike channel block by the memantine moiety, this second allosteric effect requires several minutes of drug addition for onset of action (and therefore is not evident in the short-term additions in Fig. 1) and survives washout by many minutes^20^,^27^,^28^. To insure that NitroMemantine had not entered the channel during the washout period, as opposed to during subsequent agonist addition as expected of an open-channel blocker, we analyzed the rise times of the individual NMDA-evoked responses before and after YQW-036 addition; we found that rise times were similar (inset in Fig. 2c), as expected if the antagonist were not already residing in the channel, i.e., trapped, prior to agonist addition^10^,^29^. We can further distinguish the redox effect of the nitro group from the channel block of the aminoadamantane core in two additional ways. First, the effect of S-nitrosylation is abrogated by systematic mutation of five cysteine residues (Fig. 2b). Importantly, prior work has shown that mutation of these same cysteine residues does not affect other properties of the NMDAR, including inhibition at the Mg^2+^/memantine binding site in the channel^20^,^30^. Note also that under ambient air conditions, as tested here, the majority of the effect is mediated by Cys399 on the GluN2A (previously designated NR2A) subunit. However, as previously demonstrated^22^, inhibition by the nitro group is markedly increased under hypoxic conditions relevant to stroke and vascular dementia; under these conditions, four other cysteine residues in GluN1 and GluN2 contribute to the S-nitrosylation effect. Second, S-nitrosylation, unlike channel block, can be prevented by prior incubation with a small molecule, sulfhydryl-reactive agent like methanethiosulfonate ethylammonium (MTSEA), which reacts with the same cysteine residues and thus occludes the effect of the nitro group (Fig. 2c–e).

### Targeting the NO group to the NMDAR via the memantine moiety

Memantine binding in the NMDAR-associated channel is largely abrogated by mutation at or near the Mg^2+^ site, e.g., in NMDARs with GluN1(N616R)/GluN2 subunit composition^23^,^31^. We tested the ability of the core aminoadamantane structure to target NO_x_ to the S-nitrosylation/redox sites external to the memantine-binding site in the channel by mutating the memantine binding site. Since mutation of the channel caused smaller currents than those of wild-type (WT) NMDARs, it was necessary in these experiments to use relatively high concentrations (e.g., 100 μM) of NitroMemantine in order to increase the magnitude of the effect. We compared our lead NitroMemantine drug, YQW-036, to the 1-amino-3′,5′-diethyl-7-hydroxy-adamantine scaffold without the nitro group (designated here as memantine-OH)^18^,^19^. We found that mutation of the memantine binding site largely abrogated the activity of NitroMemantines (Fig. 2f,g and Supplementary Fig. 2), whereas a high concentration of the NO donor S-nitrosocysteine (SNOC) inhibited both the WT and mutant NMDARs (Supplementary Fig. 2). These results indicate that the aminoadamantane core moiety can target the nitro group to the receptor; mutation of the aminoadamantane binding site prevented this targeting.

**Figure 3:**
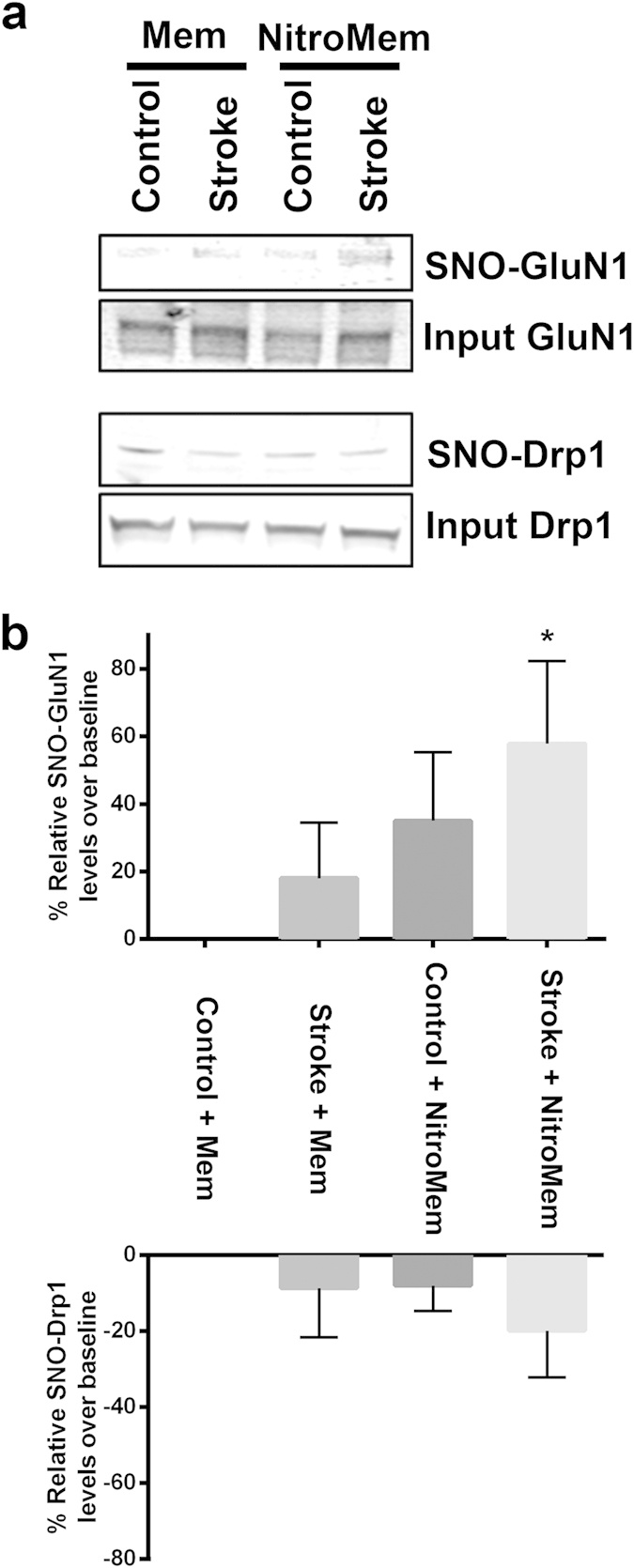
S-Nitrosylation is targeted to the NMDAR by NitroMemantine. (**a**) Spontaneously hypertensive rats (SHR) were injected with a loading dose of saline, vehicle, memantine (Mem) or NitroMemantine (NitroMem) 2-h post occlusion, and then sacrificed 90 min thereafter. Brain lysates were prepared and biotin switch assays performed to detect SNO-GluN1 and SNO-Drp1 from both the ipsilateral (stroke) and contralateral (non-stroke) hemispheres. (**b**) SNO-protein levels are presented as the ratio of ipsilateral to contralateral results above the baseline control value (mean + s.e.m.; *n* ≥ 4 for each value, *P < 0.03 compared to control by *t*- test).

To further confirm this targeting, we performed biotin switch assays to measure S-nitrosylated proteins following systemic administration of NitroMemantine YQW-036 in rats undergoing stroke. NitroMemantine, but not memantine, resulted in a significant increase in S-nitrosylation of the GluN1 subunit of the NMDAR (forming SNO-GluN1) in the cortical parenchyma ipsilateral but not contralateral to the stroke (Fig. 3). In contrast, S-nitrosylation of other proteins that we have shown to be aberrantly nitrosylated during neurodegeneration, e.g., dynamin related protein 1 (Drp1), were not significantly affected by NitroMemantine. This experiment shows pharmacodynamic targeting of the nitro group by NitroMemantine to NMDARs at the site of the pathophysiological insult.

### NitroMemantine relatively spares synaptic activity, long-term potentiation, and Morris water maze performance

**Figure 4:**
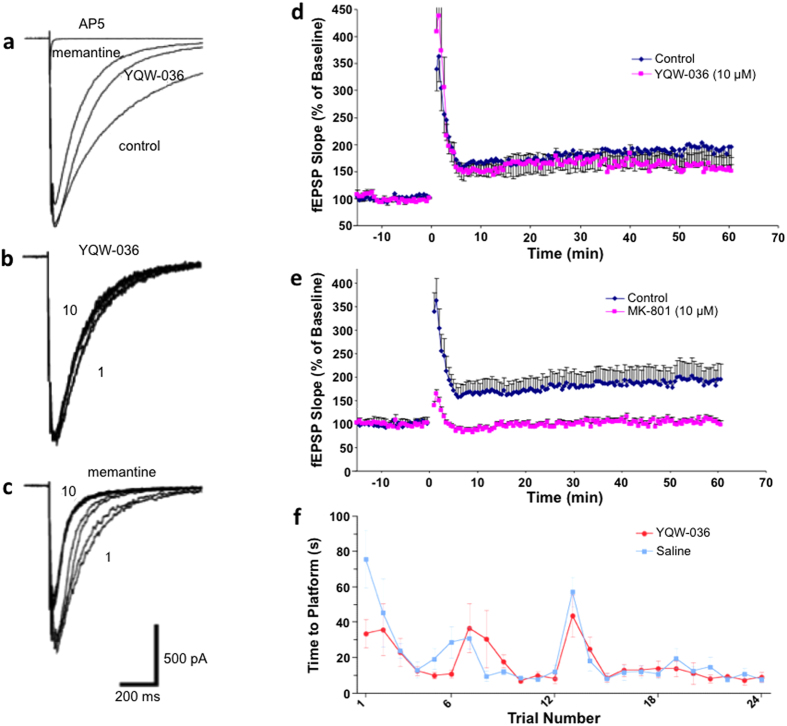
Lack of effect of NitroMemantine on EPSCs, LTP, and Morris water maze. (**a**) Whole-cell recordings of evoked EPSCs from hippocampal autapses. The NMDAR-mediated component of the EPSC was blocked to a lesser extent by 10 μM NitroMemantine YQW-036 than by 10 μM memantine. The AMPA component of the EPSC has been blocked with 20 μM CNQX. As a control, 50 μM (2*R*)-amino-5-phosphonopentanoate (d-AP5), a competitive antagonist of the glutamate binding site, completely blocked the NMDAR component of the EPSC. (**b**) The degree of blockade of the EPSC did not significantly increase with repeated electrical stimulation (pulses 1–10) in the presence of NitroMemantine YQW-036. (**c**) The inhibitory effect of memantine was use-dependent with the 10^th^-elicited EPSC being blocked more than the 1^st^. Hence, equimolar memantine blocks neurotransmission to a greater degree than NitroMemantine. For each panel, data are representative from *n* ≥ 4 patch-clamp recordings. (**d**) Neuroprotective dose of NitroMemantine (NitroMem, 10 μM) did not inhibit LTP in hippocampal CA1 induced by high-frequency burst in rat hippocampal slices. (**e**) As a control in the LTP experiments, MK-801 (equimolar, 10 μM) completely inhibited LTP induction (*n* ≥ 4 slices for panels d and e). (**f**) Lack of effect of NitroMemantine on Morris water maze performance. Water-maze testing of adult male spontaneously hypertensive rats (SHR) treated with NitroMemantine or vehicle. Rats were acclimated to the water maze on day 1, and then tested on the following 4 days, with 6 trials/day for a total of 24 trials. The platform position was changed every day, and release points for each trial varied in a pseudorandom manner. All values are mean ± s.e.m.; *n* = 5 for each group. The peaks at trials 7 and 13 represent the result of the first trial on days 2 and 3 of testing, and indicate that the animals exhibited some degree of ‘forgetfulness’ for the task overnight and had to re-learn. By day 4 (trial 19), the animals learned the new position of the platform more rapidly.

Most NMDAR antagonists produce neurobehavioral abnormalities by blocking normal neurotransmission; however, memantine does not exhibit these side effects^9^,^11^,^12^,^32^,^33^. Previously, we had tested memantine and NitroMemantine drugs on the rat hippocampal autapse preparation and on human induced pluripotent stem cell (hiPSC)-derived cortical neurons, and showed that they preferentially blocked extrasynaptic NMDAR-mediated responses, which contributed to neurodegeneration^7^,^13^,^18^. We reported that long-term addition of NitroMemantine was more effective than equimolar memantine in this regard^18^. Here, we show that NitroMemantine YQW-036 (10 μM) spared physiologic synaptic activity to a greater degree than memantine (Fig. 4a–c). Additionally, NitroMemantine spared long-term potentiation (LTP) (Fig. 4d,e), representing augmentation of synaptic currents in response to repeated synaptic inputs, which is thought to be an electrical correlate of learning and memory. Also, similar to memantine^34^, the Morris water maze, a neurobehavioral test for spatial learning and memory, was not affected by neuroprotective doses of NitroMemantine (Fig. 4f)^18^,^19^,^35^.

### Lack of histological apoptotic damage after NitroMemantine treatment

Another well-known concern over NMDAR antagonists is their propensity to cause histological damage in certain regions of the brain. For example, high-affinity NMDAR antagonists like MK-801 cause neuronal apoptosis, particularly in the developing rodent brain^36^,^37^. However, we found that neither memantine nor NitroMemantine induced neuronal apoptosis in the developing cerebrocortex at clinically-relevant dosage (Supplementary Fig. 3).

### Histological protection and neurobehavioral improvement with NitroMemantine after cerebral ischemia

**Figure 5:**
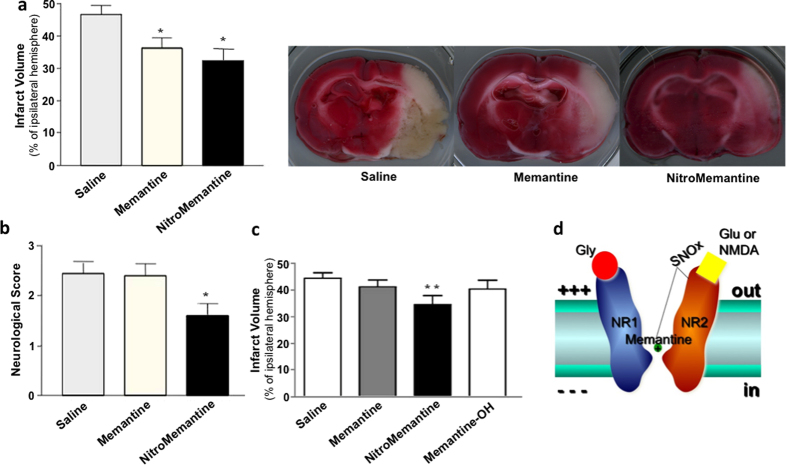
NitroMemantine protection and mechanism of action. (**a**) NitroMemantine and memantine offer histological protection in the rat tMCAO/R model. Loading doses of drug (or saline control) were administered 2-h post occlusion, maintenance doses 12 h later, and animals sacrificed at 24 h. Left: A lower dose of YQW-036 (see text) protected to a greater extent than memantine, as assessed by 2,3,5-triphenyl-2H-tetrazolium chloride (TTC) staining (*P < 0.05 by ANOVA with posthoc Scheffé). Right: Representative TTC-stained coronal brain sections under each treatment protocol. (**b**) NitroMemantine but not memantine showed a protective effect on objective neurological/behavioral testing (see Experimental Procedures, *P < 0.05 by ANOVA with posthoc Scheffé). Number of Spontaneously Hypertensive Rats (SHR) tested in panels a and b: *n* = 9 for saline group; *n* = 4 for memantine-treated group; *n* = 5 for NitroMemantine-treated group. (**c**) NitroMemantine treatment reduced the size of infarct compared to memantine, memantine-OH, and vehicle controls (**P < 0.01 by ANOVA with posthoc Scheffé). Number of SHR tested: *n* = 13 for saline group; *n* = 10 for memantine-treated group; *n* = 8 for NitroMemantine-treated group; *n* = 7 for memantine-OH-treated group. Values are mean ± s.e.m. for each panel. (**d**) Schematic of NitroMemantine action. The adamantane moiety provides targeted delivery of an NO_x_ group (where x = 1 or 2) to the NMDAR, providing two sites of antagonist action. First, an adamantane, such as memantine, enters and binds preferentially to excessively open NMDAR-coupled channels. Second, an NO_x_ group reacts with the redox site(s), comprised of reactive thiol group(s), outside of the voltage field of the channel (modified from ref. 12).

Next, we tested the ability of the lead NitroMemantine drug YQW-036 vs. memantine to offer neuroprotection using a rat model of focal cerebral ischemia consisting of transient middle cerebral artery occlusion/reperfusion (tMCAO/R), a model we and others have detailed previously^34^,^38^,^39^,^40^. Note we purposely chose a very severe model of cerebrovascular disease such that these animals died in less than two days if not treated; hence, to allow comparison, treated and untreated animals were assessed both behaviorally and histologically one day after stroke. Initially, we tested a full dose-response of drug concentration and time of delivery after insult in order to obtain a therapeutic window post stroke. To obtain significant neuroprotection, we found that we could delay treatment for up to two (but not three) hours after occlusion using the maximal feasible doses (MFDs). Hence, for the present experiments, we used the previously-determined MFD for memantine as the loading dose (90.3 μmol/kg or 20 mg/kg)^14^,^34^, administered intraperitoneal (i.p.) at the time of reperfusion (i.e., 2-h post occlusion). For NitroMemantine, only ~70% of this loading dose was injected (65.8 μmol/kg) due to limits of drug solubility. Thereafter, the standard maintenance dose of 4.63 μmol/kg (1 mg/kg) memantine was administered every 12 h^14^,^34^, whereas the dose of NitroMemantine was maintained at 70% (3.29 μmol/kg) of the memantine maintenance dose. Both drugs afforded a significant degree of histological protection after focal ischemia (Fig. 5a). Importantly, however, only NitroMemantine produced improvement in functional neurobehavioral tests affecting motor performance, even though the NitroMemantine MFD was lower than the memantine MFD (Fig. 5b). (Note: physiological parameters, including cranial and rectal temperatures, blood pressure, and metabolic panel, were not significantly affected by the loading dose of memantine or NitroMemantine (Supplementary Table 1)).

Next, in order to permit direct comparison, we tested equimolar concentrations of memantine, NitroMemantine, and its degradation product, memantine-OH, using the MFD of NitroMemantine. We observed that memantine and memantine-OH afforded no significant protection at this dosage, whereas NitroMemantine offered significant benefit on histological analysis (Fig. 5c). Taken together, these findings are consistent with the notion that NitroMemantine offers a superior outcome over memantine for focal cerebrovascular insults and that this added benefit may be attributable to targeted delivery of the nitro group, affording improved allosteric regulation of the NMDAR.

## Discussion

In the present study, we describe the mechanism of action of NitroMemantine, which provides dual-site, hypoxia-regulated antagonism at the NMDAR (Fig. 5d). The features of NitroMemantine that may confer benefit in ischemic neurons are several fold: 1. Voltage-dependent blockade of excessively open, predominantly extrasynaptic NMDAR channels; 2. Targeted-delivery of the nitro group to NMDARs by the memantine scaffold; and 3. Allosteric regulation of channel S-nitrosylation by hypoxia. Targeting of the nitro group likely occurs because open-channel blockers spend an increased proportion of their time near the channel mouth, thereby increasing the statistical odds of the nitro group reacting with vicinal cysteine residues. NitroMemantine thus modulates the biophysical parameter of “gain” of channel activity by providing two allosteric “volume controls” on the NMDAR^41^, represented by memantine action in the channel and S-nitrosylation outside of it. In this manner, excessive extrasynaptic NMDAR activity can be more effectively downregulated by NitroMemantine than by memantine alone^11^,^18^,^20^,^23^,^32^.

How S-nitrosylation of the channel occurs precisely is unclear. Alkyl nitrates such as NitroMemantine do not nitrosate directly, nor will they spontaneously release NO. However, they will form thionitrates in reaction with thiols that may rearrange to form nitrosating sulfenylnitrites. In addition, the cysteine network in the receptor may facilitate the reductive chemistry that is required^21^,^27^,^42^. Ultimately, since the site of action of the nitro group is external to the voltage field of the channel, the redox contribution to NMDAR antagonism does not decrease as the neuron depolarizes^20^,^22^,^28^. Thus, NitroMemantine can downregulate excessive NMDAR activity at a point when memantine alone would loose effectiveness.

It might be expected that the nitro group would be targeted to the NMDAR only during periods of open-channel block by the aminoadamantane moiety, i.e., in the presence of agonist. Interestingly, however, we observed a redox-mediated effect of NitroMemantines even when drug was added for a prolonged period in between applications of NMDA agonist. These results are understandable based on the known physical chemistry of aminoadamantanes, which have a very high lipid-to-water partition coefficient (see Supplementary Information in ref. 35 and citations therein). Our observed decrease in water solubility of NitroMemantine over memantine shows that lipophilicity is increased even further when the nitro group is added. Hence, aminoadamantane nitrates appear to remain in the lipid membrane like a reservoir, poised to enter the channel as soon as it opens and then also initiate the redox effect^35^. We can see that YQW-036 has not entered the channel prior to NMDA application because of the similar rise time of agonist-induced currents before and after antagonist application (Fig. 2c, inset). If the aminoadamantane nitrate were already in the channel prior to NMDA application (i.e., trapped in the closed channel), it would have slowly come out immediately upon agonist application, producing a slower rise time of NMDA-evoked current^10^,^29^. Of further note, when a high concentration of YQW-036 was used (100 μM rather than 1 to 10 μM), the lipophilic effect of the drug was further magnified, as evident in slow washout of a component of the inhibitory effect upon repeated agonist applications (Fig. 2f). Nonetheless, the fact that the prolonged effect of YQW-036 was largely abrogated by prior addition of MTSEA or mutation of redox-active cysteines in the NMDAR indicates that this component of the drug’s inhibitory action cannot be attributed solely to increased lipophilicity. Rather, the major component of the prolonged inhibitory effect of YQW-036 is best explained by interaction at the redox site(s) on the NMDAR because it was prevented by both chemical and molecular interventions at the cysteine residues that underlie redox modulation of the receptor^20^. Moreover, the finding that mutation of the principal binding site of the aminoadamantane moiety in the channel (GluN1(N616R)) largely abrogated the prolonged inhibitory effect of YQW-036 (Fig. 2g) is consistent with the notion that the memantine-like action of the drug serves to target the nitro group to the receptor. We cannot rule out the additional possibility that S-nitrosylation by NitroMemantine also increases the receptor’s susceptibility to channel block via the adamantane moiety. Moreover, the lipophilic properties of the aminoadamantane nitrates should be beneficial clinically in that they increase the concentration of drug permeating the blood-brain-barrier; in fact, due to their lipophilicity, aminoadamantanes have been reported to be concentrated at least 20-fold in brain over plasma levels (see citations in ref. 35).

We had previously published evidence that memantine does not accumulate in synaptic NMDAR-associated channels due, at least in part, to its relatively fast off-rate; hence, memantine does not adversely affect the NMDAR-mediated component of excitatory postsynaptic currents (EPSCs), NMDAR-dependent induction of LTP, or behavioral tests such as the Morris water maze^10^,^23^,^32^. The fact that memantine mostly spares synaptic/phasic activity while blocking extrasynaptic/tonically-active NMDARs is thought to underlie its clinical tolerability and reasonable clinical efficacy in human clinical trials^11^,^12^,^13^. Therefore, our finding that NitroMemantine spares even more synaptic activity while antagonizing extrasynaptic activity to an even greater degree than memantine may account for the new drug’s increased efficacy and bodes well for its clinical tolerability in human testing^18^. Additionally, hypoxic potentiation of receptor inhibition, as seen with NO-based compounds^22^, is a particularly desirable feature in ischemia. Thus, hypoxia-regulated, allosterically-targeted NMDAR antagonists, although of low affinity, may hold promise for cerebrovascular and other neurodegenerative disorders^18^,^19^.

## Methods

### Vertebrate Animals

All protocols for use of vertebrate animals were approved by the Sanford-Burnham-Prebys Medical Discovery Institute IACUC and all experiments followed ARAC guidelines. Other methods and any associated references are found in the online version of the paper.

### Chemistry

We synthesized adamantane nitrates using published reactions. Synthetic schemes and procedures are described in the Supplementary Methods.

### Oocyte recordings of NMDAR-mediated currents under two-electrode voltage clamp

Two-electrode voltage clamp recordings at −60 or −70 mV were made on oocytes in frog Ringer’s solution two to seven days after injection of NMDAR subunits. Recordings were performed with an Oocyte Clamp OC-725b amplifier (Warner Instrument Corporation, Hamden, CT) using MacLab version 3.5 software (AD Instruments, Milford, MA) at room temperature. Current-injecting electrodes had a resistance of 0.5–1 MΩ and voltage-sensing electrodes had a resistance of 1–4 MΩ. Both electrodes were filled with 3 M KCl. A solution containing 90 mM NaCl, 1 mM KCl, 10 mM HEPES, 1.5 mM BaCl_2_, and 10–100 μM glycine, pH 7.5 was used to continuously superfuse the oocytes. Barium, rather than calcium, was used as the divalent cation in order to minimize secondary activation of Ca^2+^-activated Cl^−^ current^43^. In some experiments, 1.2 mM MgCl_2_ was added to the bath solution. Drugs were dissolved in frog Ringer’s solution and applied by superfusion at a 2 ml/min flow rate. In some cases, to achieve relatively rapid solution exchange, we used an array of pipettes similar to the “sewer pipe” system used in patch-clamp recording^44^. Perfusion artifacts were digitally removed from the traces.

### Rat cerebrocortical cultures, hippocampal autapse preparation, and recordings of NMDAR-mediated currents under whole-cell clamp

Established methods were used to prepare primary rat cerebrocortical cultures and hippocampal autaptic cultures^45^,^46^,^47^. Briefly, for rat cortical cultures, cerebrocortices were dissected from embryonic day 16–17 (E16-17) Sprague-Dawley rats. After dissociation, cells were plated on poly-l-lysine-coated dishes in DMEM plus Ham’s F12 and 10% heat-inactivated iron-supplemented calf serum. Cultures were incubated at 37 °C in a humidified atmosphere of 5% CO_2_/95% air for at least three weeks prior to use to insure that their full repertoire of NMDARs were expressed. For autaptic cultures, on postnatal day 0 (P0), rat primary hippocampal cells were harvested, and a single-cell suspension prepared and plated on dot-shaped primary rat astrocyte microislands. The microislands were prepared by plating a single-cell suspension of astrocytes on collagen (62.5 μg/ml)/poly-d-lysine (50 μg/ml) microdots on agarose-coated glass coverslips. For cortical cultures, whole-cell recordings were generally performed on neurons after superfusion with 1 μM tetrodotoxin (TTX) to eliminate spontaneous activity in the cultures. For autaptic cultures, whole-cell recordings were obtained from single neurons growing on micro-islands of one or more astrocytes. Recordings were performed at room temperature on cultures 14–26 days *in vitro* (DIV) with a patch-clamp amplifier (Axopatch 200B or MultiClamp 700A Molecular Devices, Union City, CA). Cultured neurons were superfused at a rate of 2 ml/min with HEPES-buffered external solution (in mM): NaCl, 146; KCl, 2.5; CaCl_2_, 2; HEPES, 10; d-glucose, 20; pH 7.4 (NaOH adjusted), 300–310 mOsm. Borosilicate glass capillaries (GC150F-10, Warner Instruments) were used to pull patch pipettes using a micropipette puller (P87, Sutter Instruments, Novato, CA); open tip resistances were 2–7 MΩ with internal solution (in mM): CsCl, 140; NaCl, 4; CaCl_2_, 0.5; HEPES, 10; Na-GTP, 0.5; Mg-ATP, 2; EGTA, 5; pH 7.33 (CsOH adjusted); 315 mOsm. Some experiments used a Cs-gluconate based intracellular solution (in mM): Cs-gluconate, 117; NaCl, 9; HEPES, 10; and MgCl_2_, 2; EGTA, 10; pH 7.2 (NaOH adjusted). NMDAR-mediated currents were recorded in the absence of extracellular magnesium and with 10–20 μM glycine. Drugs were administered by a fast valve-controlled perfusion system (Lee Company, Essex, CT). Unless otherwise noted, all antagonists were purchased from Tocris Bioscience (Ellisville, MO). Data were acquired and analyzed as filtered and digitized electrical signals using PClamp v.10 software (Axon Instruments, Union City, CA). Data were displayed and analyzed using the statistical software package Origin 7 (OriginLab Corporation, Northampton, MA).

### Site-directed mutagenesis of NMDAR subunits

Mutant NMDAR subunits were prepared using the Chameleon^TM^ double-stranded site-directed mutagenesis kit (Stratagene) according to the manufacturer’s instructions. The entire coding region was sequenced to verify the mutation.

### Water maze neurobehavioral testing

Adult male spontaneously hypertensive rats (SHR) were treated with either PBS or NitroMemantine (65.8 μmol/kg loading dose, i.p., followed by a 3.29 μmol/kg maintenance dose every 12 h). Each group was comprised of five rats (*n* = 5). Treatment for both groups commenced 48 h prior to the first day of testing. Both the treatment and water-maze testing were conducted in a blinded fashion. The water maze consisted of a circular pool 150 cm in diameter and 85 cm deep. The pool was filled to cover a moveable platform, black in color, by 1 cm of water. The water was made opaque via the addition of black paint and maintained at room temperature (20–23 °C) for the duration of the five days of testing. A white pool with black water allowed the video camera to successfully track the white rats with maximum contrast. On day 1, the rats were placed in the pool with no platform for 60 s of acclimation. Days 2 through 5 were used for testing. Starting on day 2 and continuing to day 5, rats were trained for 24 total trials (six trials/day) to locate and reach the submerged platform. The platform was kept in the same location for all trials in each session (day), but changed from session to session in a pre-determined order, yielding four unique locations in all. In addition, there were four possible release locations (the four cardinal compass directions at the pool’s perimeter). The six trials per session/day for each animal were performed in pairs from different locations, and all animals were tested before proceeding to another pair of trials. Furthermore, the order of drop locations was constant for all rats, but, like the platform, had a pseudo-random order that changed from session to session. Each water-maze testing session was conducted in a continuous manner, without any major time gaps between individual trials or rounds of trial pairs. The maximum allowed time for the rat to successfully reach the platform was 120 s. If the animal did not reach the platform in that time, it was manually guided and given a time of 120 s. Between the two trials, the rat stayed on the platform for a minimum of 5 s (this time period excluded in determining the duration of the test).

### Assessment of rat hippocampal LTP by field recordings

Adult rats (275–300 g) were sacrificed by decapitation. Brains were rapidly removed from the skull and placed in cold artificial cerebrospinal fluid (ACSF), in mM: NaCl 116; KCl 5.37; NaHPO_4_ 1.02; NaHCO_3_ 26.19; CaCl_2_ 3.2; MgSO_4_ 0.8; d-glucose 10, pH 7.4, saturated with bubbled 95% O_2_/5% CO_2_, at 6 °C. Hippocampi were sliced at a 40-μm thickness by a manual tissue chopper (Stoelting). Slices used were from the middle third of the hippocampus, oriented in a plane perpendicular to the septotemporal axis. Slices were transferred in cold ACSF to a flow-through interface chamber and superfused at 33.5 °C. ACSF and the air interface were saturated with 95% O_2_/5% CO_2_ bubbled gas. After a 30-min incubation, the fluid was exchanged for fresh ACSF with or without MK-801, memantine or NitroMemantine, and incubated for another 60 min prior to electrophysiological recording.

Extracellular field excitatory postsynaptic potentials (EPSPs) were recorded in the stratum radiatum of the CA1 region of the hippocampus after stimulation of the CA3 Schaffer collaterals according to our previously published protocols^34^. Recordings were performed with glass microelectrodes filled with ASCF (resistance 1–2 MΩ); bipolar tungsten electrodes (Fred Haer) were used for stimulation. Responses were monitored with an AC amplifier (AM Systems). Recording electrodes were placed in the stratum radiatum at an approximately 100 μm depth under the surface and 1000 μm from the stimulating electrode. Field EPSPs were evoked and an input/output curve generated to determine the stimulus intensity required to induce a half-maximal response. Subsequent stimuli were delivered at this intensity. Paired pulse stimuli were delivered at interstimulus intervals from 6 to 200 ms, and for each interval, the degree of paired-pulse inhibition or facilitation calculated. Slope values of EPSPs were used in the paired-pulse testing paradigm. Only one electrode placement was made per slice to prevent injury to the slice. For 10–20 min, baseline responses were recorded every 30 s. After a period of observing a stable baseline, tetanic stimulation was applied to the slice (100 Hz burst for 1 s) and applied every 30 s for 60–90 min. Percent change in EPSP slope after tetanus was compared to pretetanus baseline. A repeated measures ANOVA was used to compare slopes from the different groups.

### Assessment of S-nitrosylation of NMDARs by biotin switch assay

Analysis of S-nitrosylation of NMDA receptors in rat brain was performed by the biotin switch assay, as previously described^48^,^49^. Free thiols in tissue lysates were blocked with methyl-methanethiosulfonate. Proteins were then precipitated with acetone and resuspended in HEN buffer (250 mM HEPES, pH 7.4, 1 mM EDTA, 0.1 mM neocuproine) plus 1% sodium dodecyl sulfate. Ascorbate was used to selectively reduce nitrosothiols in order to reform free thiol groups, which were subsequently biotinylated using 1 mM biotin-HPDP (Pierce, Rockford, IL). Parallel reactions were performed without ascorbate as a control to ensure specificity of the biotinylated bands. Biotinylated proteins were captured on streptavidin-agarose beads and analyzed by immunoblotting.

### Assessment of cerebrocortical apoptosis after memantine and NitroMemantine treatment

Seven-day-old rats were injected i.p. with vehicle, MK-801, memantine or NitroMemantine and the brains examined 24 h later by TUNEL (terminal deoxynucleotidyl transferase–mediated dUTP nick-end labeling) to detect apoptotic cells. Quantification was performed using the optical dissector and fractionator method (Cruz-Orive and Weibel, 1990). Briefly, a high-aperture objective and a counting frame (0.05 mm by 0.05 mm, dissector height 0.07 mm) were used to visualize and count neurons. Unbiased samplings were performed by randomly selecting 8 to 10 viewing fields within the counting frame at different focal levels using the optical dissector method. The density of normal neurons was determined by counting neurons in 70-μm-thick sections stained with a Nissl stain (methylene blue, azure II). Counting was performed in a blinded manner.

### Treatment and histological evaluation of transient middle cerebral artery occlusion with reperfusion (tMCAO/R)

Because of their known propensity to stroke, similar to human patients with hypertension, we used spontaneous hypertensive rats (SHR, Harlan) to induce tMCAO/R with the intraluminal suture method^34^,^38^,^39^,^40^. Male 200–300 g SHR were anesthetized with 3% isoflurane in 30% oxygen/70% nitrous oxide using an anesthetic vaporizer and flowmeter. Body temperature was maintained at 37 °C with a homeothermic temperature system. A laser Doppler monitor was used to monitor relative cerebral blood flow (rCBF) during the surgical procedure and recovery. rCBF measurements were made immediately after anesthesia, after occlusion of the MCA, just before, and immediately after withdrawal of the suture to initiate reperfusion. To initiate a stroke, a midline incision was made to expose the right common carotid, external carotid, and internal carotid arteries. The occipital artery branches of the external carotid artery were isolated and along with the terminal lingual and maxillary artery branches coagulated with an electrocautery unit. The internal carotid artery was isolated and carefully separated from the adjacent vagus nerve. The pterygopalatine artery was ligated close to its origin, two loosely tied sutures placed around the external carotid artery stump, and a microaneurysm clip applied to the external carotid artery near its bifurcation with the internal carotid artery. A small puncture opening was made in the external carotid artery, a monofilament (intraluminal suture) inserted through the opening, and the sutures tightened around the lumen containing the filament. The microaneurysm clip was removed from the external carotid artery and the monofilament gently advanced from the lumen of the external carotid artery into the internal carotid artery for a distance of ~19 to 20 mm beyond the bifurcation of the common carotid artery until the blood flow dropped (≥80% reduction by rCBF measurement). The suture around the external carotid artery stump was tightened to prevent bleeding. After 2 h, sutures were removed, the filament withdrawn and the incision sutured removed, with rCBF recovering at least 75% at reperfusion. Just prior to reperfusion, rats were administered memantine, NitroMemantine, or memantine-OH via i.p. injection in saline vehicle or an equivalent volume of vehicle alone. In initial experiments, the loading dose for each drug was 20 mg/kg, corresponding to 90.3 μmol/kg for memantine and 65.8 μmol/kg for NitroMemantine YQW-036 (the ∼30% lower dose of NitroMemantine was necessary due to its lower solubility in aqueous solution). Maintenance doses (4.63 μmol/kg of memantine and 3.29 μmol/kg of NitroMemantine, equivalent to 1 mg/kg or each) were administered 12 h after the loading dose. As demonstrated previously, this dose of memantine produces a biologically effective, neuroprotective concentration of 5–10 μM at the mouth of the NMDAR-associated ion channel^35^. In subsequent experiments, equimolar memantine, NitroMemantine YQW-036, or memantine-OH was used using the lower NitroMemantine dose described above. The rodents were sacrificed 24-h post occlusion, and the size of brain infarct was evaluated histologically by 2,3,5-triphenyl-2H-tetrazolium chloride (TTC) staining^50^.

### Behavioral assessment for neurological impairment after stroke

Each adult animal underwent a neurologic evaluation^51^ immediately after filament removal and again 1 day after reperfusion (prior to sacrifice). Each animal was assigned an objective behavioral score of 0–4: 0 representing no observable neurological deficit; 1 failure to extend the left forepaw; 2 circling to the left; 3 falling to the left; 4 cannot walk spontaneously. The scores obtained 24-h post stroke were averaged for each time point of each treatment group and presented as the neurological score.

### Statistical analyses

Values for the electrophysiological and histological experiments are presented as mean ± s.e.m. To test for statistical differences between groups, we used a Student’s *t*-test (two-tailed) for two-way comparisons and an ANOVA with post hoc testing for multiple comparisons. For behavioral testing, non-parametric tests (Mann-Whitney-U test) assessed the significance of the differences under the various conditions. A value of *P* < 0.05 was considered statistically significant.

## Additional Information

**How to cite this article**: Takahashi, H. *et al.* Pharmacologically targeted NMDA receptor antagonism by NitroMemantine for cerebrovascular disease. *Sci. Rep.* **5**, 14781; doi: 10.1038/srep14781 (2015).

## Supplementary Material

Supplementary Information

## References

[b1] Lo E. H. 2013 Thomas Willis Award Lecture: Causation and collaboration for stroke research. Stroke 45, 305–308 (2014). 24203848 10.1161/STROKEAHA.113.001269 PMC4067238

[b2] Simon R. P., Swan J. H., Griffiths T. & Meldrum B. S. Blockade of N-methyl-D-aspartate receptors may protect against ischemic damage in the brain. Science 226, 850–852 (1984). 6093256 10.1126/science.6093256

[b3] Chen H. *et al.* Oxidative stress in ischemic brain damage: mechanisms of cell death and potential molecular targets for neuroprotection. Antioxid. Redox. Signal. 14, 1505–1517 (2011). 20812869 10.1089/ars.2010.3576 PMC3061196

[b4] Dirnagl U., Iadecola C. & Moskowitz M. A. Pathobiology of ischaemic stroke: an integrated view. Trends Neurosci. 22, 391–397 (1999). 10441299 10.1016/s0166-2236(99)01401-0

[b5] Kemp J. A. & McKernan R. M. NMDA receptor pathways as drug targets. Nat. Neurosci. 5 Suppl, 1039–1042 (2002). 12403981 10.1038/nn936

[b6] Tymianski M. Emerging mechanisms of disrupted cellular signaling in brain ischemia. Nat. Neurosci. 14, 1369–1373 (2011). 22030547 10.1038/nn.2951

[b7] Hardingham G. E. & Bading H. Synaptic versus extrasynaptic NMDA receptor signalling: implications for neurodegenerative disorders. Nat. Rev. Neurosci. 11, 682–696 (2010). 20842175 10.1038/nrn2911 PMC2948541

[b8] Parsons M. P. & Raymond L. A. Extrasynaptic NMDA receptor involvement in central nervous system disorders. Neuron 82, 279–293 (2014). 24742457 10.1016/j.neuron.2014.03.030

[b9] Rogawski M. A. & Wenk G. L. The neuropharmacological basis for the use of memantine in the treatment of Alzheimer’s disease. CNS Drug Rev. 9, 275–308 (2003). 14530799 10.1111/j.1527-3458.2003.tb00254.x PMC6741669

[b10] Chen H. S. & Lipton S. A. Mechanism of memantine block of NMDA-activated channels in rat retinal ganglion cells: uncompetitive antagonism. J. Physiol. 499 (Pt 1), 27–46 (1997). 9061638 10.1113/jphysiol.1997.sp021909 PMC1159335

[b11] Lipton S. A. Paradigm shift in neuroprotection by NMDA receptor blockade: memantine and beyond. Nat. Rev. Drug Discov. 5, 160–170 (2006). 16424917 10.1038/nrd1958

[b12] Lipton S. A. Pathologically activated therapeutics for neuroprotection. Nat. Rev. Neurosci. 8, 803–808 (2007). 17882256 10.1038/nrn2229

[b13] Xia P., Chen H. S., Zhang D. & Lipton S. A. Memantine preferentially blocks extrasynaptic over synaptic NMDA receptor currents in hippocampal autapses. J. Neurosci. 30, 11246–11250 (2010). 20720132 10.1523/JNEUROSCI.2488-10.2010 PMC2932667

[b14] Chen H. S. *et al.* Open-channel block of N-methyl-D-aspartate (NMDA) responses by memantine: therapeutic advantage against NMDA receptor-mediated neurotoxicity. J. Neurosci. 12, 4427–4436 (1992). 1432103 10.1523/JNEUROSCI.12-11-04427.1992 PMC6576016

[b15] Erkinjuntti T., Roman G., Gauthier S., Feldman H. & Rockwood K. Emerging therapies for vascular dementia and vascular cognitive impairment. Stroke 35, 1010–1017 (2004). 15001795 10.1161/01.STR.0000120731.88236.33

[b16] Orgogozo J. M., Rigaud A. S., Stoffler A., Mobius H. J. & Forette F. Efficacy and safety of memantine in patients with mild to moderate vascular dementia: a randomized, placebo-controlled trial (MMM 300). Stroke 33, 1834–1839 (2002). 12105362 10.1161/01.str.0000020094.08790.49

[b17] Shih A. Y. *et al.* The smallest stroke: occlusion of one penetrating vessel leads to infarction and a cognitive deficit. Nat. Neurosci. 16, 55–63 (2013). 23242312 10.1038/nn.3278 PMC3952571

[b18] Talantova M. *et al.* Abeta induces astrocytic glutamate release, extrasynaptic NMDA receptor activation, and synaptic loss. Proc. Natl. Acad. Sci. USA 110, E2518–2527 (2013). 23776240 10.1073/pnas.1306832110 PMC3704025

[b19] Wang Y. *et al.* The pharmacology of aminoadamantane nitrates. Curr. Alzheimer Res. 3, 201–204 (2006). 16842096 10.2174/156720506777632808

[b20] Choi Y. B. *et al.* Molecular basis of NMDA receptor-coupled ion channel modulation by S-nitrosylation. Nat. Neurosci. 3, 15–21 (2000). 10607390 10.1038/71090

[b21] Lipton S. A. & Stamler J. S. Actions of redox-related congeners of nitric oxide at the NMDA receptor. Neuropharmacology 33, 1229–1233 (1994). 7870283 10.1016/0028-3908(94)90021-3

[b22] Takahashi H. *et al.* Hypoxia enhances S-nitrosylation-mediated NMDA receptor inhibition via a thiol oxygen sensor motif. Neuron 53, 53–64 (2007). 17196530 10.1016/j.neuron.2006.11.023 PMC1855274

[b23] Chen H. S. & Lipton S. A. Pharmacological implications of two distinct mechanisms of interaction of memantine with N-methyl-D-aspartate-gated channels. J. Pharmacol. Exp. Ther. 314, 961–971 (2005). 15901795 10.1124/jpet.105.085142

[b24] Blanpied T. A., Clarke R. J. & Johnson J. W. Amantadine inhibits NMDA receptors by accelerating channel closure during channel block. J. Neurosci. 25, 3312–3322 (2005). 15800186 10.1523/JNEUROSCI.4262-04.2005 PMC6724906

[b25] Silver I. A. & Erecinska M. Intracellular and extracellular changes of [Ca2^+^] in hypoxia and ischemia in rat brain *in vivo*. J. Gen. Physiol. 95, 837–866 (1990). 2163431 10.1085/jgp.95.5.837 PMC2216343

[b26] Wollmuth L. P., Kuner T., Seeburg P. H. & Sakmann B. Differential contribution of the NR1- and NR2A-subunits to the selectivity filter of recombinant NMDA receptor channels. J. Physiol. 491 (Pt 3), 779–797 (1996). 8815211 10.1113/jphysiol.1996.sp021257 PMC1158818

[b27] Lipton S. A. *et al.* A redox-based mechanism for the neuroprotective and neurodestructive effects of nitric oxide and related nitroso-compounds. Nature 364, 626–632 (1993). 8394509 10.1038/364626a0

[b28] Lipton S. A. *et al.* Cysteine regulation of protein function--as exemplified by NMDA-receptor modulation. Trends Neurosci. 25, 474–480 (2002). 12183209 10.1016/s0166-2236(02)02245-2

[b29] Blanpied T. A., Boeckman F. A., Aizenman E. & Johnson J. W. Trapping channel block of NMDA-activated responses by amantadine and memantine. J. Neurophysiol. 77, 309–323 (1997). 9120573 10.1152/jn.1997.77.1.309

[b30] Choi Y., Chen H. V. & Lipton S. A. Three pairs of cysteine residues mediate both redox and zn2+ modulation of the nmda receptor. J. Neurosci. 21, 392–400 (2001). 11160420 10.1523/JNEUROSCI.21-02-00392.2001 PMC6763802

[b31] Kotermanski S. E., Wood J. T. & Johnson J. W. Memantine binding to a superficial site on NMDA receptors contributes to partial trapping. J. Physiol. 587, 4589–4604 (2009). 19687120 10.1113/jphysiol.2009.176297 PMC2768015

[b32] Chen H. S. & Lipton S. A. The chemical biology of clinically tolerated NMDA receptor antagonists. J. Neurochem. 97, 1611–1626 (2006). 16805772 10.1111/j.1471-4159.2006.03991.x

[b33] Parsons C. G., Stoffler A. & Danysz W. Memantine: a NMDA receptor antagonist that improves memory by restoration of homeostasis in the glutamatergic system--too little activation is bad, too much is even worse. Neuropharmacology 53, 699–723 (2007). 17904591 10.1016/j.neuropharm.2007.07.013

[b34] Chen H. S. *et al.* Neuroprotective concentrations of the N-methyl-D-aspartate open-channel blocker memantine are effective without cytoplasmic vacuolation following post-ischemic administration and do not block maze learning or long-term potentiation. Neuroscience 86, 1121–1132 (1998). 9697119 10.1016/s0306-4522(98)00163-8

[b35] Okamoto S. *et al.* Balance between synaptic versus extrasynaptic NMDA receptor activity influences inclusions and neurotoxicity of mutant huntingtin. Nat. Med. 15, 1407–1413 (2009). 19915593 10.1038/nm.2056 PMC2789858

[b36] Ikonomidou C. *et al.* Blockade of NMDA receptors and apoptotic neurodegeneration in the developing brain. Science 283, 70–74 (1999). 9872743 10.1126/science.283.5398.70

[b37] Olney J. W. *et al.* Drug-induced apoptotic neurodegeneration in the developing brain. Brain Pathol. 12, 488–498 (2002). 12408236 10.1111/j.1750-3639.2002.tb00467.x PMC8095833

[b38] Gu Z. *et al.* A highly specific inhibitor of matrix metalloproteinase-9 rescues laminin from proteolysis and neurons from apoptosis in transient focal cerebral ischemia. J. Neurosci. 25, 6401–6408 (2005). 16000631 10.1523/JNEUROSCI.1563-05.2005 PMC6725288

[b39] Gu Z. *et al.* S-nitrosylation of matrix metalloproteinases: signaling pathway to neuronal cell death. Science 297, 1186–1190 (2002). 12183632 10.1126/science.1073634

[b40] Wang Y. F. *et al.* Tissue plasminogen activator (tPA) increases neuronal damage after focal cerebral ischemia in wild-type and tPA-deficient mice. Nat. Med. 4, 228–231 (1998). 9461198 10.1038/nm0298-228

[b41] Lipton S. A. Turning down, but not off. Nature 428, 473 (2004). 15057811 10.1038/428473a

[b42] Ischiropoulos H. *et al.* Peroxynitrite-mediated tyrosine nitration catalyzed by superoxide dismutase. Arch. Biochem. Biophys. 298, 431–437 (1992). 1416974 10.1016/0003-9861(92)90431-u

[b43] Leonard J. P. & Kelso S. R. Apparent desensitization of NMDA responses in Xenopus oocytes involves calcium-dependent chloride current. Neuron 4, 53–60 (1990). 1690016 10.1016/0896-6273(90)90443-j

[b44] Vyklicky L. Jr., Benveniste M. & Mayer M. L. Modulation of N-methyl-D-aspartic acid receptor desensitization by glycine in mouse cultured hippocampal neurones. J. Physiol. 428, 313–331 (1990). 2172523 10.1113/jphysiol.1990.sp018214 PMC1181649

[b45] Bekkers J. M. & Stevens C. F. Excitatory and inhibitory autaptic currents in isolated hippocampal neurons maintained in cell culture. Proc. Natl. Acad. Sci. USA 88, 7834–7838 (1991). 1679238 10.1073/pnas.88.17.7834 PMC52398

[b46] Segal M. M. Epileptiform activity in microcultures containing one excitatory hippocampal neuron. J. Neurophysiol. 65, 761–770 (1991). 1646871 10.1152/jn.1991.65.4.761

[b47] Segal M. M. & Furshpan E. J. Epileptiform activity in microcultures containing small numbers of hippocampal neurons. J. Neurophysiol. 64, 1390–1399 (1990). 2283535 10.1152/jn.1990.64.5.1390

[b48] Jaffrey S. R. & Snyder S. H. The biotin switch method for the detection of S-nitrosylated proteins. Sci. STKE 2001, pl1 (2001). 11752655 10.1126/stke.2001.86.pl1

[b49] Uehara T. *et al.* S-nitrosylated protein-disulphide isomerase links protein misfolding to neurodegeneration. Nature 441, 513–517 (2006). 16724068 10.1038/nature04782

[b50] Bederson J. B. *et al.* Evaluation of 2,3,5-triphenyltetrazolium chloride as a stain for detection and quantification of experimental cerebral infarction in rats. Stroke 17, 1304–1308 (1986). 2433817 10.1161/01.str.17.6.1304

[b51] Mackensen G. B. *et al.* Neuroprotection from delayed postischemic administration of a metalloporphyrin catalytic antioxidant. J. Neurosci. 21, 4582–4592 (2001). 11425886 10.1523/JNEUROSCI.21-13-04582.2001 PMC6762378

