# Corrigendum: Pharmacologically targeted NMDA receptor antagonism by NitroMemantine for cerebrovascular disease

**DOI:** 10.1038/srep20750

**Published:** 2016-02-12

**Authors:** Hiroto Takahashi, Peng Xia, Jiankun Cui, Maria Talantova, Karthik Bodhinathan, Wenjun Li, Sofiyan Saleem, Emily A. Holland, Gary Tong, Juan Piña-Crespo, Dongxian Zhang, Nobuki Nakanishi, James W. Larrick, Scott R. McKercher, Tomohiro Nakamura, Yuqiang Wang, Stuart A. Lipton

Scientific Reports 5: Article number: 14781; 10.1038/srep14781 published online: 10192015; updated: 02122016.

Sofiyan Saleem was omitted from the author list in the original version of this Article. This has been corrected in the PDF and HTML versions of the Article.

The Author Contributions section now reads:

S.A.L. designed and supervised all experiments and prepared the manuscript. Y.W. and J.W.L. performed chemical synthesis of aminoadamantane nitrates, analysis of the structure-activity relationship, and hit-to-lead optimization. H.T., P.X., M.T., T. T, J.P.-C., K.B. and D.Z. performed or analyzed electrophysiological experiments. J.C. and S.S. performed the surgery for the animal model of cerebral ischemia with assistance of W.L. T.N. performed the pharmacodynamic assay showing S-nitrosylation of the NMDA receptor with assistance of E.H. S.R.M. and N.N. helped analyze data, performed statistical analysis, prepared figures, and assisted in experiments. S.A.L. and S.R.M. wrote the manuscript, and all coauthors participated in editing the manuscript.

