# Supplementary material for: Pharmacologically targeted NMDA receptor antagonism by NitroMemantine for cerebrovascular disease
Source: Sci Rep. 2015 Oct 19;5:14781. doi: 10.1038/srep14781 (PMC4609936; doi:10.1038/srep14781)
Supplement: Supplementary Information [file srep14781-s1.pdf]

Scientific Reports

Supplementary Information:

Pharmacologically targeted NMDA receptor antagonism by NitroMemantine for cerebrovascular disease

Hiroto Takahashi, Peng Xia, Jiankun Cui, Maria Talantova, Karthik Bodhinathan, Wenjun Li, Emily Holland, Gary Tong, Juan Piña-Crespo, Dongxian Zhang, Nobuki Nakanishi, James W. Larrick, Scott R. McKercher, Tomohiro Nakamura, Yuqiang Wang & Stuart A. Lipton

Supplementary Information

Supplementary Methods

Supplementary Figures and Table

Supplementary Methods

Chemical synthesis. The aminoadamantane nitrate **[8]**, designated 'NitroMemantine' YQW-036, was synthesized as illustrated in **Scheme 1**, below. Commercially available 3, 5-diethyladamantane underwent a Koch-Haff reaction by treatment with concentrated sulfuric acid and formic acid to produce the acid **[2]** with a 59% yield, as described previously[52]. The acid **[2]** was brominated by treatment with dry bromine and anhydrous aluminium bromide at 0-10 °C to produce **[3]** (67% yield) using a previously reported method[53]. Compound **[3]** was first treated with ethyl chloroformate and then with sodium azide to afford the carboxyazide compound **[4]**[54]. Without further purification, compound **[4]** was hydrolyzed with 10% hydrobromic acid to afford the amino alcohol **[5]** (20% yield). The amino group of the latter was then protected by treatment with di-*tert*-butyl dicarbonate [$(BOC)_2O$] to generate compound **[6]** (71% yield). Nitration was accomplished by treating compound **[6]** with acetic nitrate at 0 °C for 10 min, affording product **[7]** with a 66% yield. The BOC protective group was removed by treatment with anhydrous hydrogen chloride in ethyl acetate, affording 'NitroMemantine' YQW-036 **[8]** (95% yield). Details of each reaction step are described below.

3, 5-Diethyl-1-adamantanecarboxylic acid [2]. To fuming sulfuric acid (15 ml), cooled to 0 ^{o}C in an ice-bath, 1, 3-diethyladamantane **[1]** (1 g, 5.21 mmol) was added dropwise over 1 h. The reaction mixture was stirred for 2 h at 0 ^{o}C. Formic acid (3 ml) was then added dropwise over 1 h. The reaction was allowed to continue for another 2 h at 0 ^{o}C. The reaction mixture was then poured onto ice (100 g) slowly with vigorous stirring. The white precipitate formed was filtered and washed with water to give a crude product (1 g), which was purified by crystallization from a solution of methanol and water (CH_3OH/H_2O, 3.5/1) to give 0.73 g (59%

yield) of a white solid product, mp 65-66 ^{o}C. ^{1}H NMR (DMSO-d6, ppm): 12.03 (s, 1H, COOH), 2.07 (s, 1H), 1.64 (s, 2H), 1.46-1.26 (m, 8H), 1.61-1.06 (m, 6H), 0.78-0.74 (t, 6H, J = 7.2 Hz, 2 x CH3). Anal. ($C_{15}H_{24}O_2$) C, H.

3-Bromo-5, 7-diethyl-1-adamantanecarboxylic acid [3]. To a mixture of dry bromine (2.54 ml) and anhydrous aluminum bromide (1.52 g), cooled to 0 ^{o}C under nitrogen in a 15 ml three-neck flask, was added **[2]** (1 g, 4.24 mmol) bit by bit over a period of 1 h at 0 ^{o}C while stirring. The reaction was permitted to continue for 24 h at 0-10 ^{o}C, and then at room temperature for an additional 5 h. The reaction mixture was then poured onto 15 g of crushed ice, and the product was extracted with benzene (20 ml). The surplus bromine was reduced by reaction with solid sodium sulfite until the color of the bromine completely disappeared. The organic phase was separated, and the aqueous phase was extracted with benzene (40 ml x 2). The combined organic phase was washed subsequently with water (20 ml x 3) and a solution of 0.5 N sodium hydroxide (20 ml x 3). The alkali solutions were combined and washed with ether (20 ml). The aqueous solution was then acidified using 2 N sulfuric acid. The precipitate was filtered and dried. The product was purified by crystallization from a methanol solution (CH_3OH/H_2O, 4/1) affording product **[3]** as a white solid (0.9 g, 67 % yield), mp 109 ^{o}C. ^{1}H NMR (DMSO-d6, ppm): 12.34 (s, 1H, COOH), 2.25 (s, 2H), 1.97-1.90 (q, 4H, J = 11.99, 17.59 Hz), 1.51-1.42 (m, 4H), 1.24-1.10 (m, 6H), 0.79-0.76 (t, 6H, J = 7.6 Hz, 2 x CH3). Anal. ($C_{15}H_{23}BrO_2$) C, H.

1-Amino-3, 5-diethyl-7-hydroxyadamantane hydrobromide [5]. To product **[3]** (1.8 g, 5.71 mmol) in an acetone solution (acetone:11.4 ml; water: 2.3 ml) cooled to 0 ^{o}C, was slowly added triethylamine (0.86 g) in 14 ml of acetone, followed by ethyl chloroformate (0.81 ml, 8.53 mmol) in 14 ml of acetone. The reaction mixture was stirred for 30 minutes at 0 ^{o}C. Sodium azide (0.764 g, 11.8 mmol) in 3 ml of water was added dropwise. The reaction mixture was then

stirred for 2 h at room temperature and was poured into water (10 ml). The product was extracted with ether. The ethereal solution was washed with an 8% sodium bicarbonate solution followed by water and dried over sodium sulfate. Solvent was removed *in vacuo* at a temperature below 30 ^{o}C to yield the carboxyazide **[4]** as a white solid. Without further purification, the carboxyazide **[4]** was treated with 10% hydrobromic acid (28 ml) with stirring at reflux temperature over a period of 24 h. The reaction mixture was filtered to remove the insoluble material, and the solvent was evaporated to dryness. The product was crystallized from hot methanol to afford **[5]** as a solid (0.35 g, 20 % yield), mp 301 ^{o}C. ^{1}H NMR (DMSO-d6, ppm): 7.93 (s, 2H, NH_2), 4.81 (s, 1H, OH), 1.69 (s, 2H), 1.40-1.29 (m, 4H), 1.25-1.19 (m, 8H), 1.10-0.96 (m, 2H), 0.79-0.75 (t, 6H, J = 7.6 Hz, 2 x CH_3). Anal. ($C_{14}H_{26}NO{\cdot}HBr{\cdot}0.3H_2O$) C, H.

1-*tert*-Butoxycarbonylamino-3, 5-diethyl-7-hydroxyadamantane [6]. Compound **[5]** (0.35 g, 1.15 mmol) was dissolved in a saturated sodium bicarbonate solution (10 ml), and the solution was extracted with ether (10 ml x 3). The combined organic phase was washed with water (10 ml) and dried using sodium sulfate. Solvent was removed *in vacuo* to afford **[5]** as the free amine (0.257 g). Without further purification, amine **[5]** was dissolved in tetrahydrofuran (THF). Triethylamine (0.41 ml), di-*tert*-butyldicarbonate (0.77 g, 3.53 mmol), and dimethylaminopyridine (4.6 mg) were added subsequently. The reaction mixture was stirred for 3 h at room temperature. A solution of 0.5 N sodium hydroxide (4.6 ml) was added. The reaction mixture was stirred overnight at room temperature. After triethylamine was removed *in vacuo,* ether was added. The ether solution was washed with 0.1 N HCl and brine. The solution was dried over sodium sulfate. Solvent was removed *in vacuo*. The product was crystallized on standing in ether to afford [6] as a white solid (0.263 g, 71% yield for two steps), mp 134 ^{o}C. ^{1}H NMR (DMSO-d6, ppm): 6.45 (s, 1H, NH), 4.40 (s, 1H, OH), 1.62 (s, 2H), 1.41 (s, 4H), 1.36 (s,

9H), 1.22-1.13 (m, 8H), 0.98-0.91 (m, 2H), 0.77-0.74 (t, 6H, J = 7.6 Hz, 2 x CH_3). Anal. ($C_{19}H_{33}NO_3 \cdot 0.1H_2O$) C, H, N.

1-*tert*-Butoxycarbonylamino-3, 5-diethyl-7-adamantanenitrate [7]. Cooled (0 °C) acetyl nitrate (417 μl) from a mixture of fuming nitric acid and acetic anhydride (1:1.5/v:v) was added to **[6]** (0.228 g, 0.71 mmol) in dichloromethane (5.2 ml) at 0 °C under N_2, and the reaction mixture was stirred at 0 °C for 15 min. A solution of 1 N sodium bicarbonate (26 ml) was added carefully, and the product was extracted with dichloromethane (50 ml). The dichloromethane solution was washed with water (30 ml x 3). The solution was dried over sodium sulfate. The product was then purified by thin layer chromatography, eluting with a solution of ethyl acetate and hexane (1/6, v/v) to afford **[7]** as a white solid (0.172 g, 66% yield), mp 105-106 °C. ^{1}H NMR (DMSO-d6, ppm): 6.74 (s, 1H, NH), 2.18 (s, 1H), 1.74-1.60 (m, 6H), 1.46-1.43 (m, 2H), 1.37 (s, 9H), 1.28-1.22 (q, 4H, J = 8.0 Hz), 1.14-1.05 (dd, 2H, J = 12.39, 26.39 Hz), 0.80-0.76 (t, 6H, J = 7.2 Hz, 2 x CH3). Anal. ($C_{19}H_{32}N_2O_5$) C, H, N.

1-Amino-3, 5-diethyl-7-adamantanenitrate hydrochloride [8] (designated 'NitroMemantine' YQW-036). Anhydrous hydrogen chloride in ethyl acetate (3 N, 3 ml) was added to **[7]** (158 mg, 0.43 mmol). The reaction mixture was stirred at room temperature for 30 min. The resulting precipitate was filtered, and the product was washed with ether. Compound **[8]** was obtained as a white solid (120 mg, 95% yield), mp 218 °C. ^{1}H NMR (DMSO-d6, ppm): 8.26 (s, 3H, NH), 1.78-1.70 (dd, 4H, J = 11.59, 17.99 Hz), 1.55-1.43 (dd, 4H, J = 11.99, 36.78 Hz), 1.33-1.28 (q, 4H, J = 7.6 Hz), 1.24-1.08 (dd, 4H, J = 12.79, 49.97 Hz), 0.82-0.78 (t, 6H, J = 7.6 Hz, 2 x CH_3). Anal. ($C_{14}H_{24}N_2O_3 \cdot HCl$) C, H, N.

References

52. Moore, R. E. Conversion of adamantane hydrocarbon to monools. U.S. Patent US 3,646,224 (February 1972).

53. Hermann S. and Johann M. Über Verbindungen mit Urotropin-Struktur, XXI. Herstellung und Eigenschaften von in 3-Stellung substituierten Adamantan-carbonsäuren-(1). Chemische Berichte. 1962, 95, (3), 667–672.

54. Khardin, A. P.; Gureev, N. G. and Radchenko, S. S. Curtius rearrangement in a series of adamantane derivatives. Zhurnal Organicheskoi Khimii, 1980, 16, (1), 60-62.

Scheme 1. Synthesis of memantine nitrate YQW-036

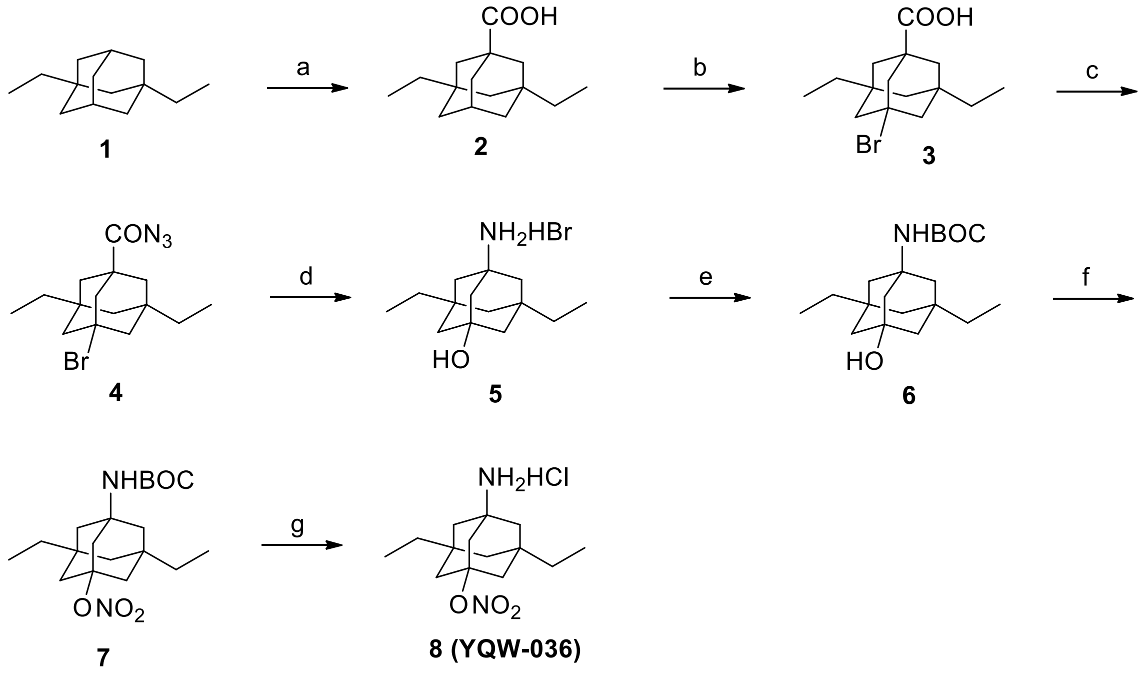

Reagents. (a) 1. fuming H_2SO_4, 2. HCOOH; (b) Br_2, $AlBr_3$; (c) 1, $ClCO_2C_2H_5$, $N(C_2H_5)_3$, 2, NaN_3; (d) HBr, H_2O; (e) 1, saturated $NaHCO_3$, 2, $(BOC)_2O$, DMAP; (f) HNO_3, Ac_2O; (g) HCl, EtOAc.

Supplementary Figures and Table

Supplementary Figure 1

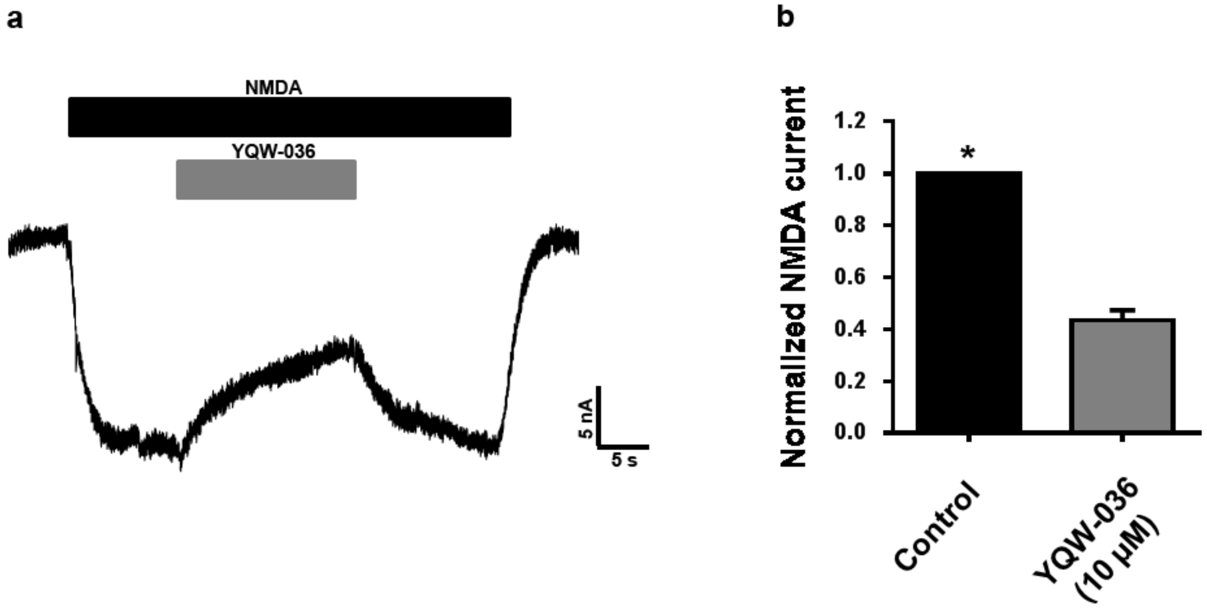

Supplementary Figure 1 | Inhibition of NMDA-evoked currents by NitroMemantine YQW-036 in the presence of extracellular magnesium ions. **(a)** Representative trace of NMDA-evoked current recorded from voltage-clamped oocytes expressing recombinant GluN1/GluN2A receptors at holding potential of -40 mV. Application of NMDA (100 μM) elicited current that was inhibited by YQW-036 (10 μM) in the presence of 1.2 mm extracellular Mg^{2+}. **(b)** Histogram of amplitude of normalized NMDA-evoked current measured at -40 mV in the presence of 1.2 mM Mg^{2+} under control conditions ($n = 6$) and in the presence of 10 μM YQW-036 ($n = 6$). Data are mean + s.e.m. (*$P < 0.01$ by Student's t-test).

Supplementary Figure 2

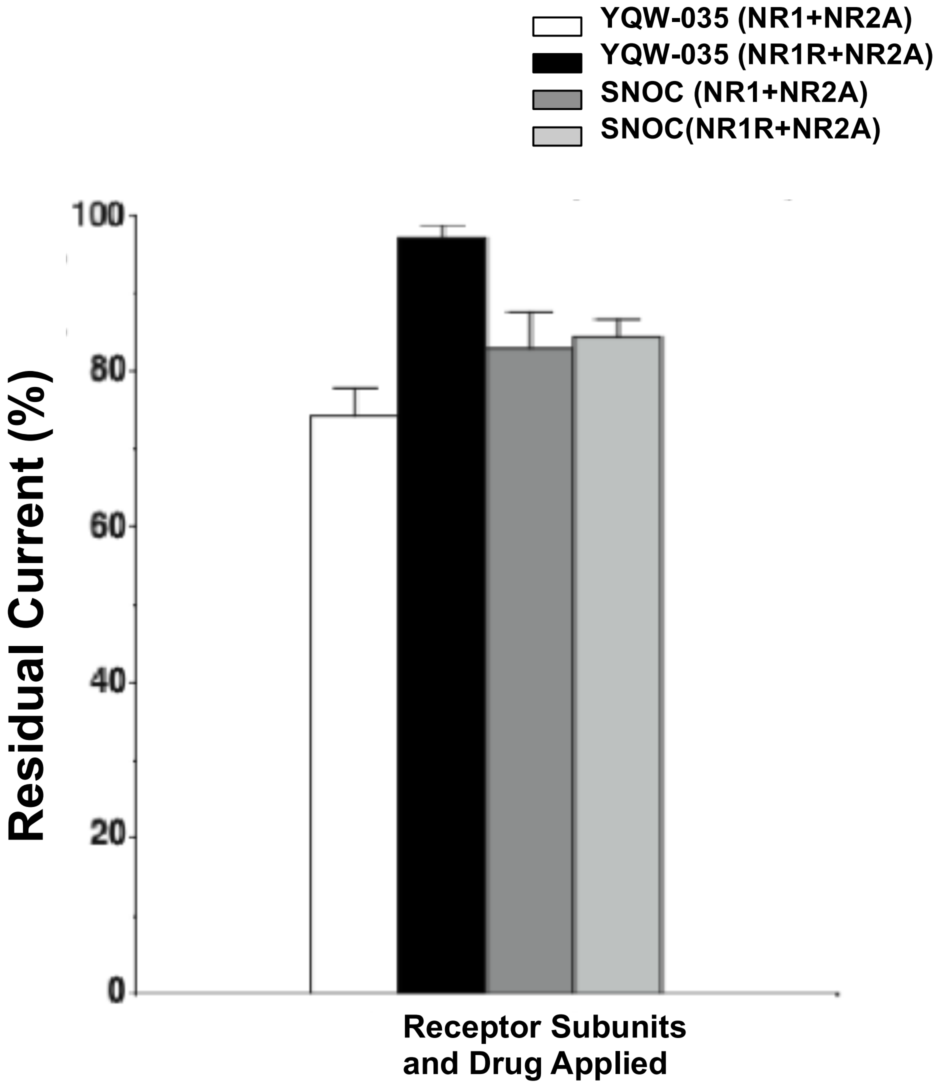

Supplementary Figure 2 | S-Nitrosylation of GluN1/GluN2A and GluN1(N616R)/GluN2A channels by S-nitrosocysteine (SNOC) or NitroMemantine YQW-035. The GluN1(N616R) mutation, which abrogates specific memantine binding in the channel, eliminated inhibition of current via S-nitrosylation by 200 µM YQW-035 but did not affect inhibition by 200 µM SNOC. Residual current represents the percentage of NMDA-evoked current remaining after inhibition by S-nitrosylation. Abbreviations: NR1 = GluN1, NR1R = GluN1(N616R), NR2A = GluN2A.

Supplementary Figure 3

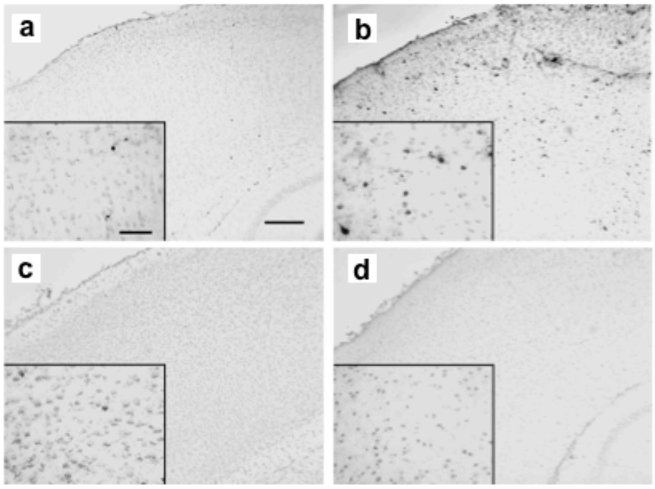

Supplementary Figure 3 | Memantine and NitroMemantine do not cause neuronal apoptosis. (a-d) Postnatal day (P)7 neonatal rat pups were injected with saline, 0.5 mg/kg MK-801, or a loading dose of memantine or NitroMemantine YQW-036 (as in Figure 7A), and then assayed 24 h later for cerebrocortical apoptosis following the published protocol of Olney and colleagues (Ikonomidou et al., 1999; Olney et al., 2002). By terminal deoxynucleotidyl transferase–mediated dUTP nick-end labeling (TUNEL) assay and morphology, MK-801 caused apoptosis **(b)**, as reported previously, but saline **(a)**, memantine **(c)**, and NitroMemantine **(d)** did not. Scale bar, 200 μm and 50 μm in inset; *n* = 3 rats for each case.

Supplementary Table 1. Physiological parameters monitored after treatment with saline, memantine, or NitroMemantine YQW-036 during stroke.

	Saline	Memantine	NitroMemantine (YQW-036)
MABP			
Before	107.00±5.29	86.63±14.08	100.20±2.23
After	104.10±5.20	86.16±8.89	100.40±2.82
pH	7.44±0.02	7.42±0.02	7.46±0.01
pCO_2 (mmHg)	35.90±1.89	39.34±1.41	32.67±1.94
pO_2 (mmHg)	173.70±8.67	188.30±12.75	169.60±3.03
Na+ (mmol/L)	144.00±0.93	141.00±1.02	144.00±0.56
K+ (mmol/L)	4.39±0.03	4.64±0.13	4.47±0.13
Glucose (mg/dl)	172.00±15.77		178.80±33.15

Physiological parameters were not significantly affected by memantine or NitroMemantine YQW-036 during stroke experiments on n = 18 SHR. MABP = mean arterial blood pressure.
